# Intersection of HIV and Anemia in women of reproductive age: a 10-year analysis of three Zimbabwe demographic health surveys, 2005–2015

**DOI:** 10.1186/s12889-020-10033-8

**Published:** 2021-01-06

**Authors:** Philimon N. Gona, Clara M. Gona, Vasco Chikwasha, Clara Haruzivishe, Chabila C. Mapoma, Sowmya R. Rao

**Affiliations:** 1 College of Nursing & Health Sciences, University of Massachusetts Boston, 100 Morrissey Boulevard, Boston, MA 02125 USA; 2 Department of Nursing, MGH Institute of Health Professions, MA Boston, USA; 3 University of Zimbabwe College of Health Sciences, Harare, Zimbabwe; 4 Department of Population Studies, University of Zambia, Lusaka, Zambia; 5 Department of Global Health, Boston University School of Public Health, Boston, MA USA

**Keywords:** Anemia in women 15–49 years, Intersection of Anemia and HIV, Zimbabwe demographic health surveys

## Abstract

**Background:**

Women of reproductive age 15–49 are at a high risk of iron-deficiency anemia, which in turn may contribute to maternal morbidity and mortality. Common causes of anemia include poor nutrition, infections, malaria, HIV, and treatments for HIV. We conducted a secondary analysis to study the prevalence of and associated risk factors for anemia in women to elucidate the intersection of HIV and anemia using data from 3 cycles of Zimbabwe Demographic and Health Survey (ZDHS) conducted in 2005, 2010, and 2015.

**Methods:**

DHS design comprises of a two-stage cluster-sampling to monitor and evaluate indicators for population health. A field hemoglobin test was conducted in eligible women. Anemia was defined as hemoglobin < 11.0 g/dL in pregnant women; < 12.0 in nonpregnant women. Chi-squared test and multivariable logistic regression analysis accounting for complex survey design were used to determine the prevalence and risk factors associated with anemia.

**Results:**

Prevalence (95% confidence interval (CI)) of anemia was 37.8(35.9–39.7), 28.2(26.9–29.5), 27.8(26.5–29.1) in 2005, 2010, and 2015, respectively. Approximately 9.4, 7.2, and 6.1%, of women had moderate anemia; (Hgb 7–9.9) while 1.0, 0.7, and 0.6% of women had severe anemia (Hgb < 7 g/dL)), in 2005, 2010, and 2015, respectively. Risk factors associated with anemia included HIV (HIV+: 2005: OR (95% CI) = 2.40(2.03–2.74), 2010: 2.35(1.99–2.77), and 2015: 2.48(2.18–2.83)]; Residence in 2005 and 2010 [(2005: 1.33(1.08–1.65), 2010: 1.26(1.03–1.53)]; Pregnant or breastfeeding women [2005: 1.31(1.16–1.47), 2010: 1.23(1.09–1.34)]; not taking iron supplementation [2005: 1.17(1.03–1.33), 2010: 1.23(1.09–1.40), and2015: 1.24(1.08–1.42)]. Masvingo, Matebeleland South, and Bulawayo provinces had the highest burden of anemia across the three DHS Cycles. Manicaland and Mashonaland East had the lowest burden.

**Conclusion:**

The prevalence of anemia in Zimbabwe declined between 2005 and 2015 but provinces of Matebeleland South and Bulawayo were hot spots with little or no change HIV positive women had higher prevalence than HIV negative women. The multidimensional causes and drivers of anemia in women require an integrated approach to help ameliorate anemia and its negative health effects on the women’s health. Prevention strategies such as promoting iron-rich food and food fortification, providing universal iron supplementation targeting lowveld provinces and women with HIV, pregnant or breastfeeding are required.

## Background

Anemia among women, especially those in their reproductive age, has been identified as a major public health problem, particularly in low and middle-income countries (LMICs) such as Zimbabwe [1]. Defined as hemoglobin of < 11.0 g/dL in pregnant women; and < 12.0 g/dL in nonpregnant women [2, 3], anemia affects half a billion women worldwide, about 29% non-pregnant and 38% pregnant, making it a global public health concern. Anemia is also associated with poor immunity, cognitive dysfunction, decreased ability to work and reduced overall quality of life [4]. Anemia causes fatigue, physical weakness and increased susceptibility to infections, and exposes pregnant women to poor maternal health and sub-optimal capacity to breastfeed and care for their newborns, infants and young children [5]. Anemia is a proxy for micronutrient deficiencies, an indicator of both poor nutrition and poor health [5–9]. Folate and iron deficiency anemia are associated with reduced productivity and increased maternal mortality [10]. The most common causes of anemia are poor nutrition (including iron, folic acid and vitamin deficiencies) and infections including Malaria and HIV. In anemia the number and size of red blood cells, or the hemoglobin concentration, falls below an established cut-off value, < 11.0 g/dL and < 12.0 g/dL in pregnant and non-pregnant women, respectively [2, 3], consequently impairing the capacity of the blood to transport oxygen around the body [5].

Reducing rates of anemia in women of childbearing age is essential to prevent low birth weight and perinatal and maternal mortality, as well as the prevalence of disease later in life [5, 10].

**Fig. 1:**
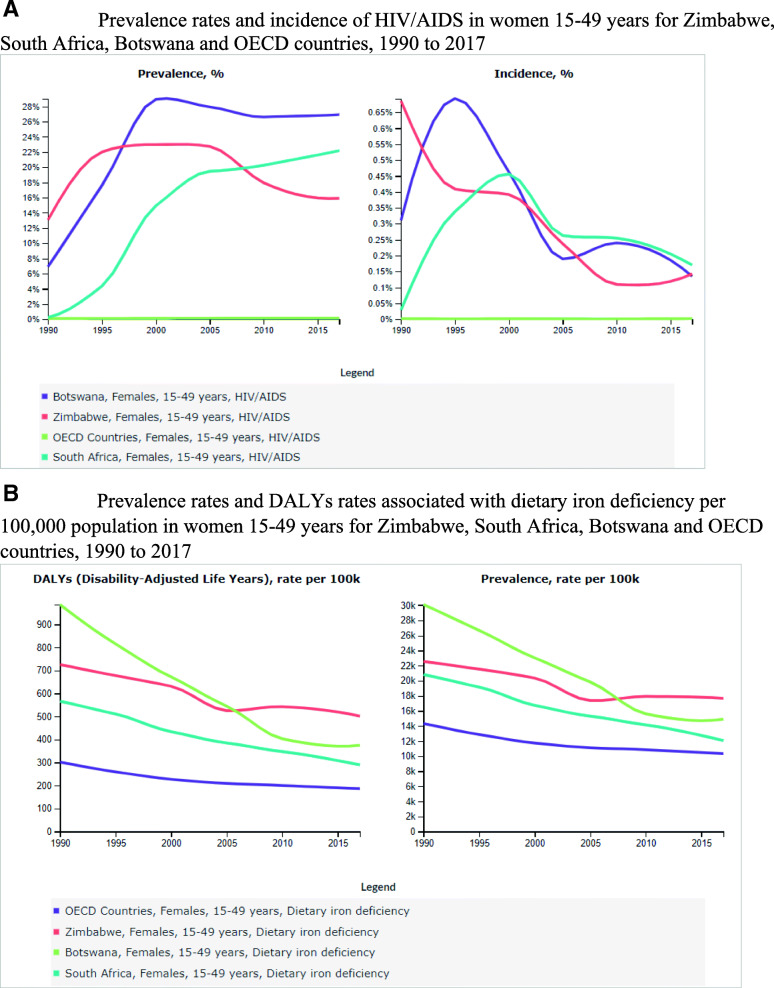
**a** Prevalence rates and incidence of HIV/AIDS in women 15–49 years for Zimbabwe, South Africa, Botswana and OECD countries, 1990 to 2017. Source: http://ghdx.healthdata.org/gbd-results-tool?params=gbd-api-2017-permalink/78b9709c512075cdeeb34d68fd02ee6b. **b** Prevalence rates and DALYs rates associated with dietary iron deficiency per 100,000 population in women 15–49 years for Zimbabwe, South Africa, Botswana and OECD countries, 1990 to 2017. Source: Top: http://ghdx.healthdata.org/gbd-results-tool?params=gbd-api-2017-permalink/2110ed1dc11a988e300e0cd36678c931

The median prevalence of anemia in pregnant women was 47.3% among the 30 LMICs for which survey data were available (2003–2015), ranging from 22.0% in Ethiopia to 75.5% in Burkina Faso [10]. In 2014 the World Health Organization (WHO) published revised guidelines to: i) support policies for the prevention and control of anemia and ii) to restore appropriate hemoglobin concentrations in individuals and reduce the prevalence of anemia in a population. Two recent resolutions by WHO and UN highlight the global importance of anemia. Firstly, Resolution 65.6 of the WHO Assembly in 2012 endorsed a comprehensive implementation plan on maternal nutrition targeting a 50% reduction of anemia in women of reproductive age by 2025 [11]. Secondly, in 2019, the UN Inter-Agency and Expert Group on the SDG Indicators [12] revised UN 2030 Sustainable Development Goal (SDG Target 2.1), (Goal 2.2) which aims to end all forms of malnutrition by 2030 [9]. One of the indicators for this Target is to create access to safe, nutritious and sufficient food all year round and reduce the percentage of women of reproductive age with anemia [11]. Since many of the drugs used to treat HIV can cause anemia, individuals with HIV infection are at increased risk for developing the condition [13, 14]. In light of the WHO and UN SDG revision of Target 2.1, Goal 2.2, and the question of the intersectionality of HIV and anemia, we were motivated to explore the prevalence of anemia in Zimbabwe, a country in the Southern Africa Development Community (SADC), where HIV/AIDS remains a massive public health threat. According to https://www.sadc.int/issues/hiv-aids/ [accessed 8/1/2020], in 2013 SADC countries accounted for nearly 3 in 4 of all people dying from HIV/AIDS-related causes. In 2010, the HIV prevalence rate in Zimbabwe was 15%. Majority of people living with HIV were women of child-bearing age as shown in Fig. 1a and b [15]. Timely and robust evidence on the burden and trends of anemia are, therefore, essential to informing policy and goal setting, program evaluation, and decision-making.

We therefore conducted a secondary analyses of data collected in the Zimbabwe Demographic and Health Survey (ZDHS) for 2005, 2010, and 2015 Cycles to: (i) determine the prevalence of and risk factors for anemia in women of reproductive age (15–49 years) in Zimbabwe, a country with a great burden of HIV [16] and (ii) in particular, examine the intersection of HIV and anemia in women of reproductive age over the 10 year duration (2000–2015).

## Methods

Demographic health surveys (DHS) are systematic and are used to collect comparable data in most LMICs. They are conducted at intervals of approximately every 5 years aiming to provide data for monitoring and evaluating indicators for population health and health information for use by policymakers, planners, researchers and program managers. These surveys are designed to yield representative information for most of the indicators for countries, by both urban and rural residencies. DHS surveys are designed to cover 100% of the selected households [17]. Detailed methods are provided in the DHS manual (https://dhsprogram.com/pubs/pdf/DHSG1/Guide_to_DHS_Statistics_DHS-7.pdf accessed on October 2, 2020).

### Sampling design

DHS uses a two-stage cluster sampling procedure with a stratified sample using probability proportional to size, of enumeration areas (EAs) selected at the first stage and a randomly selected household within the selected EAs for the second stage. All women aged 15–49 in the selected household are interviewed [17].

**Fig. 2:**
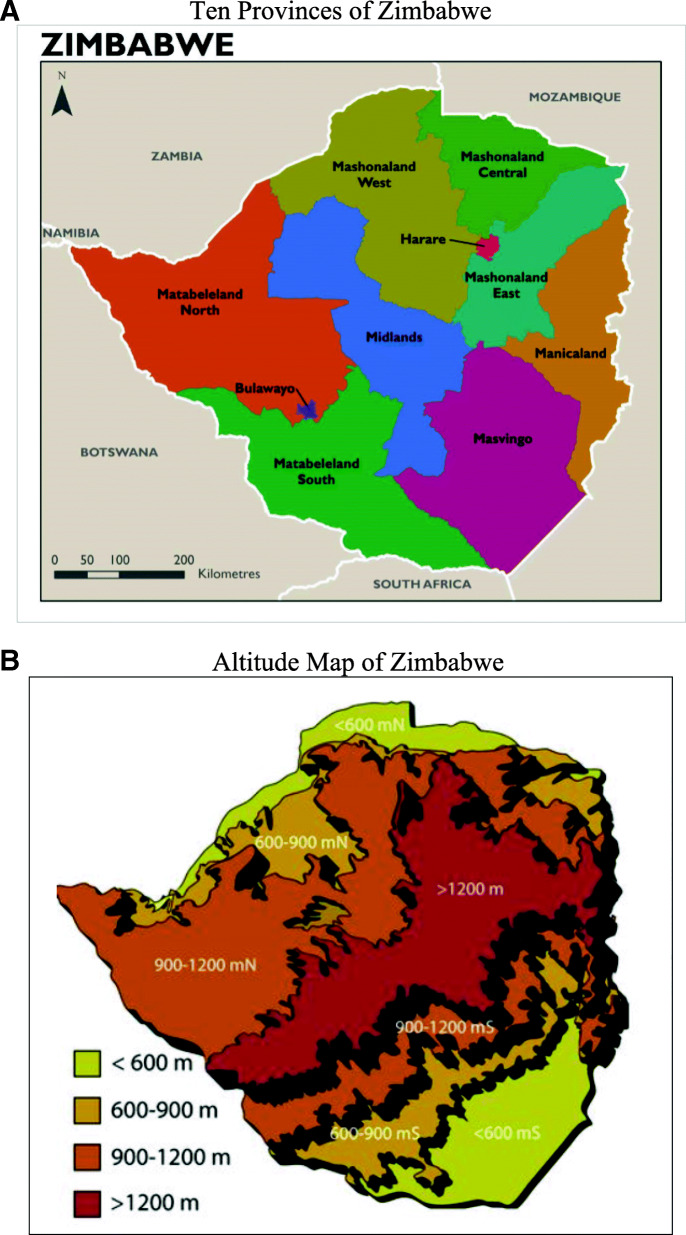
**a** Ten Provinces of Zimbabwe. Source: https://www.dhsprogram.com/pubs/pdf/SR234/SR234.pdf. **b** Altitude Map of Zimbabwe. The middle northeast to southwest belt covering the three Mashonaland provinces, part of Midlands, Matebeleland North and Matebeleland South, are in highveld (elevation > 900 m are in the highveld, the rest are in drought-prone lowveld, elevation < 600 m. Source:https://ocw.jhsph.edu/index.cfm/go/find.browse#images/topicID/16/

According to the Zimbabwe Census and Statistics Act, censuses are held every 10 years. Each DHS cycle used the most recent census of non-institutionalized population as the sampling frame excluding those living in army barracks, hospitals, police camps, and boarding schools. The DHS sample was designed to yield representative information for most indicators for the whole country, for urban and rural areas, and for each of Zimbabwe’s 10 provinces or sub-national regions: Manicaland, Mashonaland Central, Mashonaland East, Mashonaland West, Matabeleland North, Matabeleland South, Midlands, Masvingo, Harare, and Bulawayo (Fig. 2a). The three Mashonaland Provinces are in highveld, the rest are in drought-prone lowveld (Fig. 2b). Administratively each province is further divided into districts, each district into smaller administrative units called wards, and each ward into convenient areas (EAs). In 2015 DHS, 400 EAs were selected (166 in urban areas and 234 in rural areas) from a total of 29,365. A sample of 28 per EA with a typical size of 101 households were selected for a total of 11,196 private households.

### Weighting and confidentiality

Sampling weights are computed for the households and women selected for the study based on the probability of selection of each household, and response rates for households and individuals. Sampling weights are computed for the households and women selected for the study based on the probability of selection of each household, and response rates for households and individuals.

DHS computes sampling weight for a particular household as the inverse of its household selection probability multiplied by the inverse of the household response rate in the stratum and for an individual woman for a particular household as the inverse of its household selection probability multiplied by the inverse of the household response rate in the stratum [17]. Additional sampling weights are computed for sample subsets, biomarkers or HIV testing. Finally, the weights are normalized by dividing each weight by the average of the initial weights (equal to the sum of the initial weight divided by the sum of the number of cases) so that the sum of the normalized weights equals the sum of the cases over the entire sample. Using these sampling weights in the analyses allows investigators to generalize results to the target population. Furthermore, DHS has protocols in place to protect the privacy of the surveyed households and participants [17]. Detailed of the methods used to create weights are provided in the DHS manual (https://dhsprogram.com/pubs/pdf/DHSG1/Guide_to_DHS_Statistics_DHS-7.pdf accessed on October 2, 2020).

### Questionnaires

DHS surveys collect data through four main questionnaires: Household questionnaire, Woman’s Questionnaire, Men’s Questionnaire, and Biomarker Questionnaire [17]. The Household Questionnaire is used to collect information on characteristics of the household’s dwelling unit and characteristics of usual residents. It is also used to identify members of the household who are eligible for an individual interview. Eligible respondents are then interviewed based on their gender using an Individual Woman’s to obtain their characteristics including age, education, and geographic location (urban vs. rural). The Biomarker Questionnaire is used to collect biomarker data. The household roster within this questionnaire captures socio-demographic characteristics of each member. The resultant dataset filenames, variable types, names, and coding are uniform across cycles [17]. The questionnaires are updated each cycle to add new questions, but the core questionnaire is barely changed from cycle to cycle. Detailed information regarding DHS questionnaires are provided in the DHS manual (https://dhsprogram.com/pubs/pdf/DHSG1/Guide_to_DHS_Statistics_DHS-7.pdf accessed on October 2, 2020).

### Location: provinces, rural vs. urban

For each DHS cycle, the most recent population census was used as the sampling frame. According to the Zimbabwe Census and Statistics Act, censuses are held every 10 years. The 2012 Zimbabwe Population Census was used as the sampling frame for the 2015 Zimbabwe DHS. The DHS sample was designed to yield representative information for most indicators for the whole country, for urban and rural areas, and for each of Zimbabwe’s 10 provinces or sub-national regions: Manicaland, Mashonaland Central, Mashonaland East, Mashonaland West, Matabeleland North, Matabeleland South, Midlands, Masvingo, Harare, and Bulawayo. The three Mashonaland Provinces are in highveld, the rest are in drought-prone lowveld (Fig. 2a and b). Administratively each province in Zimbabwe is further divided into districts, and each district is divided into smaller administrative units called wards. Each ward was subdivided into convenient areas, which are called census enumeration areas (EAs).

In 2015 DHS there were 29,365 EAs from which 400 were selected (166 in urban areas and 234 in rural areas). A typical EA size was about 101 households from which a sample of 28 households per EA were selected for a total sample of 11,196 households [17]. The sample was selected with a stratified, two-stage cluster design, with EAs as the sampling units for the first stage. The 2015 DHS cycle sample, for example, included 400 EAs-166 in urban areas and 234 in rural areas. The second stage of sampling included the listing exercises for all households in the survey sample. A complete listing of households was conducted for each of the 400 selected EAs in March 2015 [17]. Maps were drawn for each of the clusters and all private households were listed. Sampling weight and wealth index were derived from the Households Questionnaire. The listing excluded institutional living arrangements such as army barracks, hospitals, police camps, and boarding schools.

### Outcome measures

*Anemia testing:* Anemia levels of all eligible and consented women aged 15–49 years were tested. Protocols for anemia and that for blood specimen collection for HIV testing were reviewed and approved by the Medical Research Council of Zimbabwe (MRCZ), the Institutional Review Board of ICF International, and the Centers for Disease Control and Prevention (CDC) in Atlanta, Georgia, USA. Before sample collection, verbal informed consent was obtained directly from respondents. For participants younger than 16, written and informed consent was obtained from their parent or guardian.

To be eligible for anemia test, a woman of age 15–49 years should be a usual resident or slept in the household the night before the household interview. After consenting a health specialist records all data related to biomarkers. Hemoglobin analysis is conducted immediately on the spot using a battery-operated portable HemoCue® analyzer (HemoCue AB, Angelhom, Sweden) that does not require trained phlebetomists or sample storage, producing a result in less than 1 minute [17, 18]. Testing is voluntary and respondents receive results immediately, as well as information on anemia prevention [19]. Anemia was defined as hemoglobin < 11.0 g/dL and < 12.0 in pregnant and non-pregnant women, respectively. Severe anemia was defined as < 7 g/dL; moderate 7–9.9; and mild 10–11.9 [2, 3]. All women with moderate to severe were referred for follow-up care [17]. .The Biomarker Questionnaire and Biomarker Field Manual are provided at (https://dhsprogram.com/pubs/pdf/DHSG1/Guide_to_DHS_Statistics_DHS-7.pdf accessed on October 2, 2020).

Optimal sample size for multi-stage stratified design like a two-stage cluster sampling design is dependent on intra-cluster correlation [17, 18, 20]. To estimate the prevalence of anemia for ZDHS 2015, it was assumed the background prevalence was 0.268, SE = 0.006, design effect = 1.388. A weighted sample of *n* = 9235 is enough to estimate with at least 95% confidence the prevalence of anemia to lie between 0.255–0.281, i.e., a margin of error (E) of 0.013. A similar approach was used for the DHS Cycles conducted in 2005 and 2010.

### Independent variables

*HIV Testing*: Blood specimen collection and analysis was based on the anonymous linked protocol developed for The DHS Program that allows HIV test results to be merged with the socio-demographic data collected in the individual questionnaires after the record is de-identified [17, 21]. The ZDHS biomarker interviewers explained the blood collection procedure, the confidentiality of the data, and the non-availability of the test results to the respondent.

### HIV status

HIV testing protocol provides for informed, anonymous, and voluntary testing making it difficult to provide the results to participants. Eligible women are identified on the Household Questionnaire (if using paper questionnaires) or Biomarker Data Form (if using electronic questionnaires).

### Anthropometry measurements

Height and weight were measured and body mass index (BMI; kg/m^2^) calculated.

### Household wealth index

The DHS uses Principal Component Analysis to construct the household wealth index using a composite measure of a household’s cumulative living standard based on ownership of selected assets, such as televisions and bicycles or a car; materials used for housing construction; and source of drinking water, toilet facilities, and flooring materials. (www.dhsprogram.com/programming/wealth%20index/Steps_to_constructing_the_new_DHS_Wealth_Index.pdf). The resulting asset scores are standardized in relation to a standard normal distribution with a mean of 0 and a standard deviation of 1. Individuals in the same household are assigned the same wealth index score. These standardized scores are then used to create the break points that define wealth quintiles as: Lowest, Second, Middle, Fourth, and Highest [17, 21].

#### .Statistical analysis

All analyses were conducted separately for each cycle adjusting for the complex sampling design by using the appropriate weights. We computed prevalence estimates of anemia along with 95% confidence intervals (CIs) and then conducted bivariate analyses to evaluate the association of anemia status with age in 5-year intervals (15–19, 20–24, 25–29, 30–34, 35–39, 40–44, 45–49), HIV status (positive, negative), BMI based on western standards [< 18 (underweight), 18–25 (normal weight), 25–30(overweight) and > =30 (obesity)], urban vs. rural, quintiles of household wealth index (poorest, poorer, middle, richer, richest), pregnancy/breastfeeding status (yes, no), iron consumption in pregnancy (yes, no), and province (Manicaland, Mashonaland Central, Mashonaland East, Mashonaland West, Matebeleland North, Matebeleland South, Midlands, Masvingo, Harare, Bulawayo). We computed adjusted Odds Ratios (ORs) and 95% CIs from a multivariable logistic regression model that included all the above variables. Statistical analyses were conducted with SAS/STAT (Release 9.4, SAS Institute Inc., Cary, North Carolina, USA) and Stata, Version 14.2 (StataCorp, College Station, Texas, USA). A two-sided *p*-value< 0.05 was considered significant.

## Results

### Univariate and bivariate results

Of the 7634, 8169, and 9235 women surveyed in 2005, 2010, and 2015 DHS cycles, the crude prevalence (95% CI) of anemia was 37.8(35.9–39.7), 28.2(26.9–29.5), and 27.8(26.5–29.1), respectively. The mean age for women across the 3 cycles was 28 years and 76, 74 and 72% of women in each cycle were younger than 35 years (Table 1). The prevalence of HIV decreased from 20.4% in 2005 to 16.3% in 2015. A typical woman in each cycle had a BMI of 24 kg/m^2^, however, overweight and obesity increased by 10% over the 10-years, from 25.8% in 2005 to 31.8 and 35.4% in 2010 and 2015, respectively. Underweight (BMI < 18 kg/m^2^), decreased from 5.6 to 4.1% and 3.5%, in 2010 and 2015, respectively (Table 1).

**Table 1:** Characteristics of women 5–49 years of Age in 2005, 2010 and 2015 Cycles of Demographic Health Surveys

Characteristic	2005 (***N*** = 7634)	2010 (***N*** = 8169)	2015 (***N*** = 9235)
***Age (years), Mean (95% CI)***	27.6 (27.4–27.8)	28.2 (28.0–28.4))	28.5 (28.3–28.7)
***Age: n; %(se)***			
15–19	1840; 24.1 (0.57)	1723; 21.0 (0.50)	2061; 22.3 (0.53)
20–24	1646; 21.6 (0.49)	1647; 20.2 (0.48)	1578; 17.1 (0.42)
25–29	1263; 16.5 (0.46)	1516; 18.6 (0.49)	1546; 16.7 (0.46)
30–34	1054; 13.8 (0.50)	1141; 14.0 (0.42)	1499; 16.2 (0.48)
35–39	718; 9.4 (0.44)	932; 11.4 (0.40)	1126; 12.2 (0.42)
40–44	593; 7.8 (0.34)	659; 8.1 (0.30)	896; 9.7 (0.34)
45–49	521; 6.8 (0.33)	552; 6.8 (0.31)	529; 5.7 (0.25)
***HIV Status: n; %(se)***			
Positive	1556; 20.4 (0.74)	1402; 17.2 (0.54)	1504; 16.3 (0.56)
Negative	5871; 76.9 (0.73)	6491; 79.5 (0.61)	7505; 81.3 (0.58)
***BMI (kg/m***^***2***^***), mean (95% CI)***	24.1 (23.8–24.5)	23.9 (23.7–24.0)	24.4 (24.2–24.5)
***BMI level, n; %(se)***			
< 18	424; 5.6 (0.35)	334; 4.1 (0.24)	322; 3.5 (0.22)
18- < 25	5230; 68.5 (0.71)	5225; 64.0 (0.61)	5613; 60.8 (0.71)
25- < 30	1331; 17.4 (0.63)	1761; 21.6 (0.52)	2154; 23.3 (0.52)
> =30	639; 8.4 (0.41)	833; 10.2 (0.43)	1117; 12.1 (0.46)
***Hemoglobin (g/dL), mean(95% CI)***	12.5 (12.4–12.7)	12.7 (12.6–12.8)	12.7 (12.6–12.8)
***Anemia, n; %(se)***	2882; 37.8 (0.96)	2305; 28.2 (0.65)	2474; 27.8 (0.64)
***Anemia level, n; %(se)***			
Severe	79; 1.0 (0.14)	55; 0.7 (0.11)	51; 0.6 (0.08)
Moderate	717; 9.4 (0.56)	587; 7.2 (0.34)	565; 6.1 (0.36)
Mild	2086; 27.3 (0.65)	1663; 20.4 (0.55)	1859; 20.1 (0.51)
No Anemia	4752; 62.3 (0.96)	5865; 71.8 (0.65)	6761; 73.2 (0.64)
***Wealth Index, mean(95%CI)***	3.15 (3.00–3.29)	3.17 (3.09–3.26)	3.20 (3.12–3.28)
***Wealth Index Quintile, n; %(se)***			
Poorest	1412; 18.5 (1.90)	1422; 17.4 (0.91)	1596; 17.3 (1.02)
Poorer	1347; 17.7 (0.90)	1448; 17.7 (0.72)	1589; 17.2 (0.72)
Middle	1398; 18.3 (1.20)	1540; 18.9 (1.03)	1662; 18.0 (0.85)
Richer	1666; 21.8 (1.25)	1801; 22.0 (0.91)	2141; 23.2 (1.25)
Richest	1812; 23.7 (1.68)	1959; 24.0 (1.42)	2248; 24.3 (1.36)
***Pregnant/breast-feeding, n; %(se)***	2016; 26.4 (0.84)	2382; 29.2 (0.75)	2215; 24.0 (0.60)
***Iron in pregnancy, n; %(se)***	1560; 20.4 (0.67)	2007; 24.6 (0.59)	3988; 43.2 (0.83)
***Rural/Urban Location, n; %(se)***			
Rural	4872; 63.8 (2.34)	5173; 63.3 (1.14)	5770; 62.5 (1.14)
Urban	2762; 36.2 (2.34)	2996; 36.7 (1.14)	3465; 37.5 (1.14)
***Province, n; %(se)***			
Manicaland	877; 11.5 (0.86)	1092; 13.4 (0.66)	1151; 12.5 (0.56)
Mash Central	652; 8.5 (1.61)	796; 9.8 (0.60)	848; 9.2 (0.46)
Mashonaland East	657; 8.6 (0.58)	757; 9.3 (0.72)	867; 9.4 (0.55)
Mashonaland West	696; 9.1 (0.80)	923; 11.3 (0.85)	1073; 11.6 (0.72)
Matabeleland North	470; 6.2 (0.60)	406; 5.0 (0.41)	452; 4.9 (0.25)
Matabeleland South	367; 4.8 (0.45)	429; 5.3 (0.36)	400; 4.3 (0.19)
Midlands	1127; 14.8 (0.97)	1033; 12.7 (0.79)	1177; 12.8 (1.01)
Masvingo	1046; 13.7 (2.36)	824; 10.1 (0.60)	1125; 12.2 (0.75)
Harare	1175; 15.4 (1.03)	1482; 18.1 (1.04)	1597; 17.3 (0.99)
Bulawayo	567; 7.4 (0.56)	428; 5.2 (0.31)	545; 5.9 (0.30)

The distribution of anemia status within participant characteristics in each DHS cycle is presented in Table 2. Prevalence of anemia decreased in all women and was generally higher (> 41%) in women aged 30+ years in 2005 than those under-30 (< 37%). HIV-positive women across the 3 cycles had 20% higher prevalence than HIV-negative women. There was an inverse relationship between BMI categories and prevalence of anemia. For underweight women (BMI < 18 kgs/m^2^) the prevalence decreased from 41.4% in 2005 to 28.0% in 2015, whereas in obese women (BMI > 30 kg/m^2^) the prevalence decreased from 33.6 to 23.4% for the same period. Matebeleland South and Bulawayo, and Midlands had the largest burden of anemia across the 3 cycles. Notably, while 9 provinces experienced declines in prevalence between 2005 and 2015 ranging − 5.7% to − 13.5%, the prevalence in Matebeleland South (45.0% in 2005; 44.6, 43.1% in 2015) the prevalence remained essentially unchanged. Mashonaland West (− 22.6%), Mashonaland Central (− 13.5%), Mashonaland West (− 11.9%) and Mashonaland East (− 11.3%) had > 11% declines in prevalence (Table 2).

**Table 2:** Prevalence of Anemia by characteristics for women 15–49 years of age in 2005, 2010, and 2015 cycles of demographic health surveys

Characteristic	2005, ***N*** = 7634	***p***-value	2010 (***N*** = 8169)	***p***-value	2015 (***N*** = 9235)	***p***-value
Overall, ***n; %(se)***	2882; 37.8 (0.96)		2305; 28.2 (0.65)		2474; 26.8 (0.64)	
Age, ***n; %(se)***						
15–19	634; 34.5 (1.35)	< 0.001	443; 25.7 (1.11)	0.3031	546; 26.5 (1.16)	0.1956
20–24	552; 33.5 (1.37)		470; 28.5 (1.24)		401; 25.4 (1.26)	
25–29	469; 37.1 (2.18)		439; 29.0 (1.32)		400; 25.9 (1.33)	
30–34	434; 41.3 (1.80)		318; 27.9 (1.46)		384; 25.6 (1.51)	
35–39	299; 41.7 (2.01)		271; 29.0 (1.60)		323; 28.7 (1.45)	
40–44	262; 44.2 (2.61)		200; 30.4 (1.94)		260; 29.0 (1.69)	
45–49	231; 44.3 (3.27)		164; 29.7 (2.42)		160; 30.3 (2.22)	
***HIV Status n; %(se)***						
HIV positive	841; 54.1 (1.40)	< 0.001	610; 43.5 (1.46)	< 0.001	657; 43.7 (1.76)	< 0.001
HIV negative	1949; 33.2 (1.20)		1590; 24.5 (0.69)		1759; 23.4 (0.60)	
***BMI level, n; %(se)***						
< 18	175; 41.4 (3.46)	0.0422	94; 28.3 (2.44)	< 0.001	90; 28.0 (2.60)	0.0236
18- < 25	2029; 38.8 (1.01)		1565; 30.0 (0.80)		1567; 27.9 (0.79)	
25- < 30	458; 34.4 (1.44)		455; 25.8 (1.19)		550; 25.6 (1.23)	
> =30	215; 33.6 (3.14)		184; 22.1 (1.57)		261; 23.4 (1.36)	
***Wealth Index, n; %(se)***						
Poorest	568; 40.2 (2.59)	0.0696	398; 28.0 (1.69)	0.4552	433; 27.1 (1.32)	0.0282
Poorer	460; 34.1 (1.59)		393; 27.2 (1.33)		366; 23.0 (1.22)	
Middle	507; 36.3 (1.44)		411; 26.7 (1.37)		444; 26.7 (1.38)	
Richer	668; 40.1 (1.60)		535; 29.7 (1.18)		612; 28.6 (1.38)	
Richest	679; 37.5 (1.44)		567; 29.0 (1.18)		619; 27.6 (1.00)	
***Pregnant/breast-feeding, n; %(se)***						
No	2093; 37.3 (0.90)	0.1642	1632; 28.2 (0.71)	0.9818	1901; 27.1 (0.72)	0.2981
Yes	789; 39.1 (1.59)		673; 28.2 (1.28)		573; 25.9 (1.05)	
***Iron in pregnancy, n; %(se)***						
No	2309; 38.0 (0.92)	0.3892	1789; 29.0 (0.74)	0.0159	1509; 28.8 (0.85)	< 0.001
Yes	573; 36.7 (1.74)		515; 25.7 (1.20)		965; 24.2 (0.83)	
***Location, n; %(se)***						
Urban	1074;38.9 (1.22)	0.3455	915; 30.6 (0.98)	0.0046	995; 28.7 (1.10)	0.0221
Rural	1808; 37.1 (1.37)		1389; 26.9 (0.87)		1480; 25.6 (0.78)	
***Province, n; %(se)***						
Manicaland	269; 30.7 (2.25)	< 0.001	335; 30.7 (2.10)	< 0.001	250; 21.7 (1.59)	< 0.001
Mashonaland Central	242; 37.1 (2.18)		184; 23.2 (2.55)		199; 23.5 (1.99)	
Mashonaland East	221; 33.6 (2.57)		222; 29.4 (2.06)		193; 22.3 (1.57)	
Mashonaland West	263; 37.8 (1.92)		205; 22.2 (1.54)		278; 25.9 (1.97)	
Matebeleland North	168; 35.7 (2.24)		109; 26.7 (2.39)		117; 25.9 (2.45)	
Matebeleland South	165; 45.0 (2.91)		191; 44.6 (2.35)		173; 43.1 (1.88)	
Midlands	425; 37.7 (1.84)		309; 29.9 (1.97)		367; 31.2 (2.29)	
Masvingo	496; 47.5 (3.59)		185; 22.4 (1.98)		260; 23.1 (1.64)	
Harare	418; 35.6 (1.83)		403; 27.2 (1.38)		447; 29.9 (1.78)	
Bulawayo	215; 38.0 (1.91)		162; 37.9 (2.09)		160; 29.4 (1.90)	

### Multivariable results

Table 3 shows multivariable adjusted ORs and 95% CIs for the association of anemia with participant characteristics within each DHS cycle. In 2005, there was a significant association between anemia and women in older age-group [40–44: 1.66(1.28–2.16),; 45–49: 1.80(1.35–2.41)] which did not exist in 2010 and 2015 (Table 3). When restricted to HIV negative women, the odds for anemia were elevated in 2005 [40–44: 1.71(1.34–2.20); 44–49: 1.88(1.37–2.60)]. A negative linear trend between the odds for anemia and age-groups was observed in 2005 but not in 2010 or 2015.

**Table 3:** Multivariable adjusted odds ratio for Anemia for mothers 15–49 years, 2005, 2010, and 2015 cycles of demographic health surveys

Characteristic	2005 (***N*** = 7417)	2010 (***N*** = 7879)	2015 (***N*** = 8979)
***Age-group***			
15–19	1.12 (0.91–1.39)	0.89 (0.76–1.03)	1.08 (0.87–1.36)
20–24	1.00 (referent)	1.00 (referent)	1.00 (referent)
25–29	1.09 (0.90–1.33)	1.00 (0.85–1.17)	1.05 (0.92–1.19)
30–34	1.23 (0.99–1.52)	0.89 (0.76–1.04)	0.99 (0.81–1.21)
35–39	1.32 (1.00–1.75)	0.99 (0.86–1.15)	1.08 (0.85–1.38)
40–44	1.66 (1.28–2.16)*	1.14 (0.91–1.44)	1.05 (0.81–1.36)
45–49	1.80 (1.35–2.41)***	1.07 (0.78–1.46)	1.16 (0.83–1.62)
***HIV Status***			
Positive	2.40 (2.03–2.74) ***	2.35 (1.99–2.77) ***	2.48 (2.18–2.83)***
***BMI Level***			
< 18	1.16 (0.88–1.53)	0.91 (0.69–1.19)	0.89 (0.71–1.12)
18- < 25	1.00 (referent)	1.00 (referent)	1.00 (referent)
25- < 30	0.80 (0.70–0.91)	0.75 (0.65–0.88)	0.87 (0.76–1.00)
> =30	0.68 (0.54–0.86) *	0.61 (0.52–0.72)***	0.75 (0.65–0.88)*
**Wealth Index Quintile**			
Poorest	0.93 (0.74–1.18)	1.04 (0.81–1.32)	1.04 (0.74–1.44)
Poorer	0.75 (0.55–1.03)	1.00 (0.83–1.22)	0.86 (0.65–1.15)
Middle	0.87 (0.66–1.13)	0.90 (0.72–1.12)	1.01 (0.76–1.35)
Richer	1.00 (0.83–1.21)	1.02 (0.81–1.28)	1.04 (0.90–1.19)
Richest	1.00 (referent)	1.00 (referent)	1.00 (referent)
**Pregnant/breast-feeding**	1.31 (1.16–1.47)***	1.23 (1.09–1.34)***	1.13 (0.97–1.31)
**No iron in pregnancy**	1.17 (1.03–1.33)*	1.23 (1.09–1.40) *	1.24 (1.08–1.42)**
**Location**			
Rural	1.33 (1.08–1.65)*	1.26 (1.03–1.53) *	1.01 (0.79–1.28)
Province			
Manicaland	1.00 (referent)	1.00 (referent)	1.00 (referent)
Mashonaland Central	1.36 (1.18–1.57)*	0.68 (0.56–0.81)***	1.11 (0.92–1.35)
Mashonaland East	1.11 (0.99–1.23)	0.94 (0.79–1.11)	0.99 (0.75–1.31)*
Mashonaland West	1.31 (1.11–1.54)*	0.61 (0.53–0.71)***	1.21 (1.00–1.47)
Mat. North	1.19 (1.02–1.39)*	0.75 (0.52–1.07)	1.12 (0.93–1.34)
Matebeleland South	1.86 (1.65–2.10) ***	1.70 (1.34–2.17) ***	2.46 (2.21–2.75)***
Midlands	1.36 (1.12–1.63)*	0.89 (0.77–1.04)	1.57 (1.40–1.77)***
Masvingo	2.23 (1.90–2.62)***	0.63 (0.51–0.77)***	1.02 (0.88–1.19)
Harare	0.96 (0.73–1.27)	0.70 (0.60–0.82)**	1.49 (1.21–1.84)*
Bulawayo	1.10 (0.88–1.36)	1.12 (0.91–1.39) **	1.48 (1.26–1.74)*

*p < 0.05, **p < 0.01, ***p < 0.001

Similar analyses are shown by subgroup status in Supplemental Table 1 (restricted to HIV negative women), and Supplemental Table 2 (restricted to HIV positive women). There was no association between age-group and anemia in HIV negative women in 2010 and 2015 (Supplemental Table 1). Upon restriction to HIV positive women, results show an association between anemia and age-group 20–29 [1.41(1.00–1.99)] in 2010. In 2015 results show elevated odds for anemia ranging from 43 to 57% higher in women age 25–44 years. Specifically, adjusted ORs by age-group were 1.57(1.09–2.25) for women 25–29, 1.43(1.04–1.95) for women 30–34, 1.50(1.00–2.24) for women 35–39, and 1.68(1.16–2.45) for women 40–44 (Supplemental Table 2).

#### Multivariable results by selected independent variables

### HIV status

In all three DHS cycles, HIV positive women had greater than 2-folds the odd for anemia: OR = 2.40(2.03–2.74) in 2005, 2.35(1.99–2.77) in 2010, and 2.48(2.18–2.83) in 2015 (Table 3).

### Pregnancy or breast-feeding status, iron supplementation in pregnancy, and wealth index

Pregnant or breast-feeding women had elevated odds for anemia in 2005 and 2015 with adjusted OR of 1.31(1.16–1.47) in 2005 and 1.23(1.09–1.34) in 2010 (Table 3). When restricted to HIV positive women only, the adjusted odds ratios were not elevated (Supplemental Table 2). However, when restricted to HIV negative women, the adjusted odds ratios were OR = 1.43(1.296–1.58) in 2005, and 1.28(1.15–1.43) in 2010. [peak of economic meltdown, and successive drought) (Supplemental Table 1). Not taking iron supplementation during pregnancy was associated with higher odds for anemia in all 3 cycles: OR = 1.17(1.03–1.33) in 2005, OR = 1.23(1.09–1.40) in 2010, and OR = 1.24(1.08–1.42) in 2015 (Table 3). Similarly, not taking iron supplementation elevated the odds for anemia in HIV negative women in all 3 cycles OR = 1.16(1.02–1.33) in 2005, OR = 1.22(1.07–1.39) in 2010, and OR = 1.26(1.07–1.48) in 2015 (Supplemental Table 1). We explored the interaction of anemia and taking iron supplementation with anemia in HIV positive women, the interaction term did not attain statistical significance.

### Anemia by BMI

While results presented earlier suggest negative effects observed between the prevalence of anemia and independent variables, the association between anemia and BMI were indicative of a protective balance. Being obese had a protective effect was against anemia with odds ratios for anemia of OR = 0.68(0.54–0.86) in 2005, OR = 0.61(0.52–0.72) in 2010, and OR = 0.75(0.65–0.88) in 2015, respectively (Table 3). In all the 3 cycles there was a significant negative linear trend in the odds for anemia with BMI (all 3 *p*-value for trend < 0.0001). The protective effect of obesity was also observed in both HIV negative and HIV positive women (Supplemental Table 1 and Supplemental Table 2).

### Rural vs. urban differentials

Relative to women residing in urban locations, rural women had elevated odds for anemia in 2005 and 2010 but not in 2015 with odds ratios OR = 1.33(1.08–1.65) in 2005, and OR = 1.26(1.03–1.53) in 2010 (Table 3). After stratification by HIV status, the odds ratios of anemia in 2005 and 2010 were 0R = 1.26(1.01–1.58), and OR = 1.28(1.08–1.52), respectively. For HIV positive women, the odds were more elevated in 2005 comparatively, OR = 1.71(1.17–2.49). There were no differences in rural vs. urban odds of anemia in HIV positive women in 2010 and 2015, respectively (Supplemental Table 1 and Supplemental Table 2).

### Provincial differentials

With the lowest prevalence of anemia of 30.7and 21.7% in 2005 and 2010, respectively, Manicaland was used as the referent province in the logistic regression analysis. In 2005, 6 provinces had significantly higher odds for anemia than Manicaland. The two provinces with the highest odds ratios were Masvingo, OR = 2.23 (1.90–2.62) and Matebeleland South, OR = 1.86(1.65–2.10). In 2010 the pattern flipped, and except for Matebaleland, which had higher odds for anemia, OR = 1.7-(1.34–2.17), four provinces Mashonaland Central, Mashonaland West, Masvingo, and Harare had significantly lower odds with odds ratios ranging from as low as 0.61(0.53–0.71) in Mashonaland West to 0.70(0.60–0.82) in Harare. In 2015 the pattern changed again, with four provinces having elevated odds ranging from the highest of 2.46(2.21–2.75) in Matebeleland South to 1.57(1.40–1.77) in Midlands, and the lowest being 1.48(1.26–1.74) in Harare (Table 3). Similar patterns were observed in the 3 cycles upon restricting the analysis to the HIV status of being negative (Supplemental Table 2) and/or being HIV positive (Supplemental Table 2).

## Discussion

This paper had a specific focus: to examine the prevalence of anemia and risk factors in women 15–49 years in Zimbabwe. We analyzed data from 3 cycles of DHS spanning 10 years between 2005 and 2015. Prevalence of anemia was 37.8, 28.2%, 27.8, in 2005, 2010, and 2015, respectively. HIV+ women, with the prevalence of 54.1, 43.5,43.7%, respectively, had 20% higher prevalence or greater than twice the odds for anemia across all the 3 cycles. There was an inverse relationship between BMI and the likelihood for anemia with lower BMI associated with higher prevalence. Provinces in the drought-prone south and south west, i.e., Matebeleland North and South, Midlands and Masvingo had higher prevalence in the 3 cycles than the highveld provinces of Mashonaland East, Central, and West; and Manicaland had lower burden of anemia.

To be effective for policymaking, these indicators for anemia need to be available at spatially granular levels such as districts or wards to best aid resource targeting such as a 5-by-5 km grid [22]. It has been shown with HIV that geographic targeting of resources can improve the efficiency and effectiveness of interventions [22, 23]. Estimates of these indicators at this fine spatial scale can assist in setting targets and tracking progress towards meeting the WHO nutrition target to achieve a 50% reduction of anemia in women of reproductive age by 2025 (https://www.who.int/nutrition/publications/globaltargets2025_policybrief_overview/en/),or meeting the revised UN 2030 Sustainable Development Goal (SDG Target 2.1). (Goal 2.2).

### Previous studies

The high anemia rate among women of child-bearing age is similar to findings from other LMICs. A meta-analysis reported an anemia rate of 42.7% (7.0, 48.4%) among pregnant women in LMICs [24, 25]. The 2015 burden of anemia in Zimbabwe (25.9% overall and 26.8% in pregnant or breastfeeding mothers) was substantially lower than the 39 and 46% (13.1 and 19.2% lower), respectively reported globally in 2016 [5]. A 2009 study of adult women across 40 DHSs from 32 countries found that although anemia rates do decrease as income increases, the decrease is modest [25]. We purposely did not account for education. While education and health infrastructure probably contribute to reduce the burden of anemia, such factors are likely to be positively correlated with wealth index. In our study, there was no statistically significant linear trend across quintiles of household Wealth Index. There was little or no difference in the prevalence of anemia between the lowest and highest quintiles of wealth index across the 3 cycles. This observation is reinforced by the assertion that even with a robust economy, it takes decades of sustained income growth to double national per capita income [25].

### .Findings in relation to SDG goals

Zimbabwe will also fall short of the revised UN 2030 Sustainable Development Goal (SDG) Target 2.1 Goal 2.2 which aims to end all forms of malnutrition by 2030 (the UN Inter-Agency and Expert Group on the SDG Indicators) [12]. With only 5 years left to attain Resolution 65.6 (before 2025), and compounding this with the current prevalence of 27.8% (and 43.7% for HIV+), Zimbabwe will have to achieve declines equivalent to 2.8% (4.4% for HIV+) annually if the Global Nutrition Target is to be met. The flattening of prevalence experienced between 2010 and 2015 does not suggest this level of annual decline to be achievable or indeed sustainable; with this in mind, there could be other possible explanations for the stagnation in prevalence of anemia between 2010 and 2015.

### Intersection of anemia prevalence with burden of HIV

Firstly, the 2005 to 2015 DHS cycles overlapped a period during which Zimbabwe was among the top 5 countries in SADC most burdened with HIV/AIDS [16]; it is also a period camouflaged by an extension of a serious economic downturn. Figure 1a shows 27-year trends of prevalence and incidence of HIV/AIDS in women 15–49 years for Zimbabwe, South Africa, Botswana and OECD countries, 1990 to 2017. Figure 1b shows the rates of prevalence and DALYs associated with dietary iron deficiency per 100,000 population in women 15–49 years for Zimbabwe, neighboring South Africa and Botswana, and the Organization for Economic Co-operation and Development (OECD) countries; between 1990 and 2017 iron deficiency anemia declined for the three countries and OECD countries, the prevalence and DALYs rates per 100,000 for Zimbabwe flattened after 2005, a pattern that mirrors our study findings. The patterns of HIV burden for Zimbabwe was a sharp contrast with the burden for Botswana and South Africa. The prevalence for Zimbabwe flattened at about 20% between 1995 and 2005 but increased for the neighboring countries. Similarly, incidence progressively declined in Zimbabwe from ~ 0.65% to ~ 0.1% unlike for Botswana and South Africa. Figure 1a and b suggest that Zimbabwe, unlike its neighbors and OECD countries, was entangled in an intersection of increasing to decreasing levels of HIV/AIDS burden coupled with the stagnant burden of dietary iron deficiency – a situation critical in giving context to our current study findings. Anti-retroviral treatment (ART) has improved survival and prevention of mother-to-child transmission of HIV. However, individuals with HIV infection are significantly more likely to develop anemia independently of HIV treatment [13]. Moreover, other studies suggest, many drugs used to treat HIV can also cause anemia [13, 14]. .In Zimbabwe, most individuals, 79% (75–83%) with HIV were on antiretroviral therapy (ART), a 30% increase, 49% (42–57%) from 2009. Note that the burden of HIV/AIDS in OECD countries was negligible (Figs. 1a and b). Our study showed that women 15 to 45 years who were HIV+, regardless of their breast-feeding or pregnancy status, had greater than 2.35 the odds for anemia than HIV- women. Our findings, taken together with the GBD study [26], provide associative but not causal evidence of elevated risk of anemia in HIV+ women. HIV status-specific analysis results shown in Supplemental Tables 1 and 2 revealed elevated odds for anemia for all characteristics between HIV positive and HIV negative women. Women 40–49 years of age without HIV have higher odds for anemia than similar women with HIV; women with BMI < 30 kg/m^2^ with HIV had higher odds for anemia than similar women without HIV; household wealth index was a predictor for anemia in women without HIV but not in women with HIV; current pregnancy or breastfeeding, and not taking iron supplementation were protective in women without HIV but not significantly so for women with HIV. For policy, the findings in Supplemental Tables 1 and 2 suggest that screening for anemia by measuring hemoglobin level should be prioritized for all women especially if they have HIV. Furthermore, leaner women with BMI < 30 kg/m^2^ with HIV should equally be prioritized for hemoglobin level screening for anemia.

### Drugs for treating HIV

Taken together, the above may explain our findings. Whether the HIV/anemia association is mediated by ART should be studied further. Since DHS 2005, 2010, and 2015 cycles not collect data on ART, we had no way to explore whether indeed the HIV/anemia association has been mediated by the ART factor. We therefore recommend, that future DHS cycles develop IRB modalities for providing individuals tested for HIV with their test results and immediately link those with positive results with appropriate care. Once a woman tests positive, it is logical to also offer the test to their spouse and if negative, the couple is offered information on how to live together as a discordant couple. Furthermore, notwithstanding that our study, because of its observational nature, is insufficient to establish causality of HIV and anemia, we recommend that all individuals with HIV should be provided iron supplementation as prevention for anemia. Pharmaceutical industries are encouraged to develop a once weekly, or once monthly iron-supplement tablet to keep the pill burden low for HIV positive individuals who could also be on ART – which, as pointed out earlier, do cause anemia [13, 14].

### Climate change

SADC countries experience more intense droughts linked to changes in El Niño/La Niña-Southern Oscillation patterns. This climate change pattern, that occurs across the tropical Pacific Ocean approximately every 5 years cause food shortages and food insecurity. Droughts occur annually in Zimbabwe. The three Mashonaland Provinces are in highveld, the rest are in drought-prone lowveld [27]. “see elevation map at: https://ocw.jhsph.edu/index.cfm/go/find.browse#images/topicID/16)”. The frequency and intensity of droughts have increased but severity and spatial distribution varies by province affecting livelihoods of the people largely because of the Zimbabwean economy’s dependence on agriculture [27]. The socio-economic impacts of drought are varied, ranging from famines, and food insecurity, caused by crop failure which could explain the high burden of anemia we observed [28]. The western regions, Matabeleland North and South Provinces, as well as Midlands provinces, with average rainfall less than 650 mm annually, are more likely to be affected by severe droughts. Droughts range from severe to mild, with average rainfall greater than 650 mm annually in the three Mashonaland provinces (East, West, and Central) to the north, and Masvingo province in the South East. In a drought year, the rain in these regions allow some form of crop production that is adapted to drought conditions like small grains [27]. Zimbabwe’s climate is projected to become more erratic, with some projections suggesting that widespread crop failure will occur every three out of 5 years. United States Agency for International Development (USAID) 2020 Climate Risk Profile Zimbabwe (2020) https://www.climatelinks.org/sites/default/files/asset/document/2020_USAID_ATLAS_CRP-Zimbabwe.pdf) The Zimbabwean government has been relying on food aid and food redistribution [27]. Between 1991 and 2016, Zimbabwe experienced six moderate-to-severe droughts. The drought severity in Zimbabwe between 2005/2006 and 2009/2010 seasons, the 2007/2008 agricultural season was the “worst” (i.e., had the largest proportion of the country under drought) whilst the 2008/2009 agricultural season was the “best” (lowest proportion of the country under drought) [27]. These patterns match the high prevalence of anemia we observed in 2005 and the steep decline observed in the two later cycles.

### Political and economic conditions

Zimbabwe experienced the first hyperinflation of the twenty-first century for the period 2006–2010. At the peak (2008–2009) of this sharp economic downturn, Zimbabwe’s inflation was estimated at 79.6 billion percent month-on-month, 89.7 sextillion percent year-on-year by mid-November 2008 [29]. .In the history of the world, only Hungary (1946) had ever experienced such a worse stratospheric inflation [29]. Such inflation, primarily caused by political instability, created chaos in all spheres of the economy, food became scarce and health outcomes suffered substantially [30–32]. Other external parameters such as the Zimbabwe Democracy and Economic Recovery Act of 2001, (ZIDERA) enacted into law by the Senate and House of Representatives of the United States of America in Congress (www.congress.gov/bill/115th-congress/senate-bill/2779/text) reversed any gains made in health and stifled affected countries from attaining the UN-led SDGs [32]. On paper, such economic sanctions ideally meant to target politicians, as our study and others alike show, have deeper and far-reaching health consequences and unnecessary loss of life affecting common people instead [31, 32].

### Summary

The simultaneous tri-factor of a) adverse climate changes causing more frequent droughts, b) high prevalence of HIV infection and most people with HIV on ART, and c) political and economic sanctions created maelstrom favoring increased propensity for anemia among women of child-bearing age. Climate change caused food shortage and food insecurity leading to malnutrition; high prevalence of HIV infection created demand of HIV drugs some of which are known to trigger anemia; and a tough economic environment worsened by economic sanctions caused the healthcare system to collapse; all of which jointly generated a vortex for anemia.

#### Strengths

Among the several strengths of our study is the representativeness of the sample. A multi-stage sampling design based on sampling units from national census and analysis accounting for this complexity allowed us to obtain well-represented population-based estimates. In addition, DHS studies have high participation rates, for example, the participation rate was 99% in 2010 ZDHS cycle, limiting the potential for participation bias and non-response biases. Further, use of standard questionnaires, standardized data collection, sampling procedures, uniform data structures and coding schemes from cycle to cycle are great strengths of the DHS maintaining both continuity and standardization and allowing comparability of results over time and across sub-national regions and countries. Notably, national-level estimates may obscure substantial heterogeneity at spatial scales such as provincial-level, sub-provincial or district-level, where policy decisions are made and implemented. The large size of this dataset at each cycle provided optimal power to estimate the prevalence of anemia and the effect of individual risk factors in a multivariable model, adjusting for many known confounders.

#### Limitations

The DHS has the limitations of any observational study. The cross-sectional nature of DHS limits our ability to assess causal relationships. Information regarding their characterstics on the survey was self-reported with no means to verify the veracity of the responses given by mothers, potentially introducing misclassification bias. However, we expect this bias to be small and not substantially affect our estimates of the association of HIV status and anemia. We also noted that lack of information on the duration women had HIV, the duration on ART, and the individual components of ART they were on, i.e., protease inhibitors, nucleotide reverse transcriptase inhibitors, and non-nucleotide reverse transcriptase inhibitors or secondary prophylaxis against opportunistic infections such as tuberculosis limited our scope to underscore how ART maybe a factor in the study or anemia in the population and among HIV+ women. Antitubercular therapy has been shown to be associated anemia [33]. The lack of association between anemia and current pregnancy, breastfeeding, and not taking iron supplementation in women with HIV contrary to women without HIV is counterintuitive. It is likely that our study was under-powered to detect these associations in women with HIV, therefore additional research is warranted. Another limitation is that there have been attempts lately to change the WHO hemoglobin cutoff points of < 11.0 g/dL and < 12.0 g/dL in pregnant and nonpregnant women, respectively [2, 3]. Any changes in cutoff points may have a tremendous effect on the prevalence of anemia presented [34]. To illustrate, our study observed prevalence of 54.1 (51.4–56.8), 43.5 (40.8–46.6), and 43.7 (40.3–47.1) in 2005, 2010 and 2015, respectively; a 2019 study of Ethiopian women with HIV reported an approximately similar prevalence of 40.1(33.1–47.1). However, the Ethiopian study used a higher cutoff point for anemia of < 13.0 g/dL making our prevalence incomparable to the Ethiopian since higher prevalence is expected if a higher cutoff point is used [35]. By design, our study was not equipped to assess proportion of HIV-related mortality and morbidity attributable to the effects of anemia, or the proportion of anemia prevalence attributable to ART. Therefore, studies to establish population attributable fractions are needed. Biomarkers such as C-reactive protein (CRP) and alpha-1 acid glycoprotein (AGP) are used to adjust iron deficiency measures and improve estimates of anemia prevalence and causes. CRP and AGP are not collected in the current DHS and should be considered in future surveys.

### Recommendations

The WHO Global Nutrition Targets 2025, Anemia Policy Brief offers a range of recommendations and actions for policymakers to consider in achieving the global nutrition targets of 50% [5]. These recommendations range from improving the identification, measurement scaling up coverage of prevention, control and treatment activities; partnerships between state and nonstate actors for financial commitment to implement comprehensive food and nutrition policies; developing programs beyond the health sector including agriculture and education sectors; and to monitor and evaluate the implementation of anemia control programs [5]. The policy brief also provides case studies for successful presentation of anemia in Viet Nam, Venezuela, and India. Others have recommended iron and folic acid supplementation, fortification of wheat and maize flours [5, 36, 37]. Although such programs have been found to be cost-effective, there are comparatively few large-scale delivery mechanisms to reach the rural poor, who are often reliant on their own or local food production and have limited access to health services [25]. Governments should increase awareness about the benefit of adhering to consumption of nutritional diet among people living with HIV/AIDS taking ART as consuming appropriate diet helps the body in proliferating enough amounts of red blood cells and other granulocytes [35]. The food industry should invest in healthy and inexpensive food. For women with HIV taking ART, hemoglobin level should be monitored on each of the follow-up visit and appropriate action taken to combat any detected anemia [35].

While some of these strategies may have been already adopted in Zimbabwe, our findings emphasize that more expansion in terms of this policy and other developing world issues are needed. For example, the Coronavirus Disease (2019) (COVID-19) pandemic which caught the world by surprise is a challenge affecting everyone. Of the of 534 COVID-19 patients admitted to hospitals in Spain, fewer than 1% (0.92%; 95% CI: 0·39–2.14) were HIV positive suggesting that HIV-positive people appear more likely to contract the new coronavirus or to become seriously ill [38]. There is anecdotal evidence that people with sickle cell anemia are more likely to have serious complications from COVID-19. Obesity predisposes individuals with SARS-CoV-2 infection to increased severity of the disease [39]. All stakeholders, i.e., the general population government authorities, and health care professionals should recognize that obesity is a chronic disease which requires a variety of effective prevention and management strategies both in response to COVID-19 and in the longer term. A comprehensive societal approach involving effective strategies will help protect people with obesity from COVID-19 complications, prevent a further increase in the prevalence of overweight and obesity [40]. The association of anemia and COVID-19 in the presence of HIV needs further investigation.

Our study findings suggest a strong association between being HIV+ and anemia, a fact devoid from the WHO Anemia Policy [5]. Given that only 5 years are remaining before setting the next anemia targets, it would be in the interest of the next policy to consider the inclusion of HIV in addressing interventions around anemia especially for populations of women in sub-Saharan Africa where HIV is endemic and significantly higher compared to the rest of the world. Another recommendation not quite related to our study findings is about the DHS HIV protocol which is designed to not disclose HIV test results with the participant. The protocol should be revised to allow for test results to be made available to the participants. Not sharing test results can be viewed as both unethical and a missed opportunity to treat infected participants before the disease gets worse.

It is believed that about 50% of all anemia cases are due to iron deficiency [41], and other major contributors include malaria [42]; infection with bacteraemia-causing organisms [43–45]. Our findings reinforce the need for further studies to understand the impact of environmental factors on anemia burden at subnational scales to help in the design of cost-effective delivery of programs and interventions. Finally, as our results show, the state of anemia in Zimbabwe remains paradoxical: the anemia burden was lower (13.1% lower for all and 19.2% lower) than the global burden (WHO) regardless of Zimbabwe simultaneously experiencing a cocktail of events favoring increased propensity for anemia among women, all which were not experienced by other countries, at least not at the scale manifested in Zimbabwe [6, 7, 27–32].

## Conclusion

Prevalence of anemia in Zimbabwe declined between 2005 and 2015 but drought-prone provinces of Matebeleland South and Bulawayo were hot spots with little or no change. HIV positive women had higher prevalence than HIV negative women. The multidimensional causes and drivers of anemia in women require an integrated approach to help ameliorate anemia and its negative health effects on the women’s health. Prevention strategies such as promoting iron-rich food and food fortification, providing universal iron supplementation targeting lowveld provinces and women with HIV, pregnant or breastfeeding combined with monitoring for anemia, and starting treatment based on the type of anemia (folic or dietary) are required.

## Supplementary Information

**Additional file 1: Supplemental Table 1.** Multivariable Adjusted Odds Ratios for HIV negative mothers 15–49 years, 2005, 2010, and 2015 Cycles of Demographic Health Surveys. **Supplemental Table 2.** Multivariable Adjusted Odds Ratios for HIV positive mothers 15–49 years, 2005, 2010, and 2015 Cycles of Demographic Health Surveys

## Data Availability

Data that support the findings of this study are available at: https://dhsprogram.com/Data/

## Abbreviations

AGP: Alpha-1 acid glycoprotein
ART: Antiretroviral treatment
BMI: Body Mass Index
CDC: Centers for Disease Control and Prevention
CI: Confidence Interval
CRP: C-Reactive Protein
COVID-19: Coronavirus Disease
DHS: Demographic and Health Survey
EA: Enumeration Areas
g/dL: grams per deciliter
OECD: Organization for Economic Co-operation and Development
OR: Odds ratio
SDGs: Sustainable Development Goals
HIV: Human immunodeficiency syndrome
UN: United Nations
USAID: United States Agency for International Development
WHO: World Health Organization
ZIDERA: Zimbabwe Democracy and Economic Recovery Act
ZDHS: Zimbabwe Demographic and Health Survey
MRCZ: Medical Research Council of Zimbabwe
