# Supplementary material for: Intersection of HIV and Anemia in women of reproductive age: a 10-year analysis of three Zimbabwe demographic health surveys, 2005–2015
Source: BMC Public Health. 2021 Jan 6;21:41. doi: 10.1186/s12889-020-10033-8 (PMC7787417; doi:10.1186/s12889-020-10033-8)
Supplement: Supplementary file 1 — Additional file 1: Supplemental Table 1. Multivariable Adjusted Odds Ratios for HIV negative mothers 15–49 years, 2005, 2010, and 2015 Cycles of Demographic Health Surveys. Supplemental Table 2. Multivariable Adjusted Odds Ratios for HIV positive mothers 15–49 years, 2005, 2010, and 2015 Cycles of Demographic Health Surveys [file 12889_2020_10033_MOESM1_ESM.docx]

**Supplemental Table 1**: Multivariable Adjusted Odds Ratios for HIV negative mothers 15-49 years, 2005, 2010, and 2015 Cycles of Demographic Health Surveys

| **Characteristic** | | | | **2005 (N=5866)** | **2010 (N=6480)** | **2015 (N=7479)** |
| --- | --- | --- | --- | --- | --- | --- |
| ***Age-group*** | | | |  |  |  |
|  | | 15-19 | | 1.20(0.96-1.50) | 0.90(0.76-1.06) | 1.06(0.85-1.32) |
|  | | 20-24 | | 1.00 (referent) | 1.00 (referent) | 1.00 (referent) |
|  | | 25-29 | | 1.14(0.89-1.46) | 0.88(0.72-1.09) | 0.97(0.83-1.12) |
|  | | 30-34 | | 1.17(0.95-1.45) | 0.98(0.81-1.20) | 0.92(0.75-1.14) |
|  | | 35-39 | | 1.26(0.87-1.83) | 0.97(0.80-1.19) | 1.01(0.76-1.35) |
|  | | 40-44 | | 1.71(1.34-2.20)** | 1.21(0.94-1.57) | 0.91(0.67-1.24) |
|  | | 45-49 | | 1.88(1.37-2.60)*** | 1.31(0.97-1.76) | 1.20(0.85-1.70) |
| ***BMI level*** | | | |  |  |  |
|  | | | <18 | 1.03(0.70-1.51) | 0.70(0.50-0.97)* | 0.81(0.60-1.09) |
|  | | | 18-<25 | 1.00 (referent) | 1.00 (referent) | 1.00 (referent) |
|  | | | 25-<30 | 0.90(0.77-1.05) | 0.78(0.69-0.90)* | 0.90(0.76-1.06) |
|  | | | >=30 | 0.68(0.53-0.87)* | 0.61(0.51-0.74)** | 0.80(0.65-0.98)* |
| ***Wealth Index Quintile*** | | | |  |  |  |
|  | | | Poorest | 0.86(0.66-1.11) | 0.99(0.77-1.27) | 1.19(0.86-1.64) |
|  | | | Poorer | 0.71(0.52-0.97)* | 1.04(0.86-1.27) | 0.92(0.72-1.19) |
|  | | | Middle | 0.76(0.58-1.00) | 0.95(0.75-1.20) | 1.15(0.88-1.50) |
|  | | | Richer | 0.99(0.83-1.20) | 1.04(0.79-1.38) | 1.07(0.94-1.22) |
|  | | | Richest | 1.00 (referent) | 1.00 (referent) | 1.00 (referent) |
| ***Pregnant/breast-feeding*** | | | | 1.43(1.296-1.58)*** | 1.28(1.15-1.43)*** | 1.10(0.92-1.31) |
| ***No iron in pregnancy*** | | | | 1.16(1.02-1.33)* | 1.22(1.07-1.39)** | 1.26(1.07-1.48)** |
| Rural | | | | 1.26(1.01-1.58)* | 1.28(1.08-1.52)** | 1.14(0.92-1.42) |
| Province | | | |  |  |  |
|  | Manicaland | | | 1.00 (referent) | 1.00 (referent) | 1.00 (referent) |
|  | Mashonaland Central | | | 1.38(1.11-1.72)* | 0.67(0.57-0.80)*** | 1.06(0.83-1.36) |
|  | Mashonaland East | | | 1.25(1.10-1.43)* | 0.93(0.78-1.10) | 0.95(0.71-1.27) |
|  | Mashonaland West | | | 1.31(1.07-1.61)* | 0.59(0.49-0.70)*** | 1.13(0.91-1.41) |
|  | Mat. North | | | 1.21(1.02-1.44)* | 0.79(0.60-1.03) | 1.03(0.85-1.24) |
|  | Matebeleland South | | | 1.98(1.72-2.28)*** | 1.67(1.35-2.06)*** | 2.45(2.20-2.73)*** |
|  | Midlands | | | 1.37(1.09-1.72)* | 0.79(0.68-0.91)* | 1.56(1.38-1.76)*** |
|  | Masvingo | | | 2.46(2.02-2.98)*** | 0.59(0.50-0.70)*** | 1.02(0.88-1.18) |
|  | Harare | | | 1.03(0.74-1.42) | 0.66(0.55-0.79)** | 1.40(1.14-1.72)* |
|  | Bulawayo | | | 1.28(0.99-1.65) | 1.21(0.97-1.52) | 1.43(1.22-1.69)* |

*p<0.05, **p<0.01, ***p<0.001

**Supplemental Table 2**: Multivariable Adjusted Odds Ratios for HIV positive mothers 15-49 years, 2005, 2010, and 2015 Cycles of Demographic Health Surveys

| **Characteristic** | | | **2005 (N=1551)** | **2010 (N=1400)** | **2015 (N=1501)** |
| --- | --- | --- | --- | --- | --- |
| ***Age-group*** | | |  |  |  |
|  | | 15-19 | 0.78(0.47-1.31) | 1.04(0.71-1.52) | 1.08(0.55-2.13) |
|  | | 20-24 | 1.00 (referent) | 1.00 (referent) | 1.00 (referent) |
|  | | 25-29 | 0.95(0.68-1.33) | 1.41(1.00-1.99)** | 1.57(1.09-2.25)* |
|  | | 30-34 | 1.27(0.82-1.98) | 0.71(0.49-1.03) | 1.43(1.04-1.95)* |
|  | | 35-39 | 1.37(0.94-2.02) | 1.00(0.69-1.47) | 1.50(1.00-2.24) |
|  | | 40-44 | 1.53(0.92-2.53) | 0.96(0.65-1.42) | 1.68(1.16-2.45)* |
|  | | 45-49 | 1.64(0.97-2.78) | 0.60(0.37-0.97) | 1.27(0.77-2.10) |
| ***BMI level*** | | |  |  |  |
|  | | <18 | 1.72(1.10-2.71)*** | 1.84(1.10-3.09)*** | 1.15(0.67-1.96) |
|  | | 18-<25 | 1.00 (referent) | 1.00 (referent) | 1.00 (referent) |
|  | | 25-<30 | 0.52(0.35-0.76)*** | 0.65(0.46-0.92)*** | 0.83(0.63-1.10) |
|  | | >=30 | 0.74(0.51-1.08) | 0.54(0.33-0.86)*** | 0.61(0.43-0.87)* |
| ***Wealth Index Quintile*** | | |  |  |  |
|  | | Poorest | 1.32(0.70-2.50) | 1.20(0.78-1.84) | 0.59(0.24-1.47) |
|  | | Poorer | 1.00(0.59-1.69) | 0.85(0.50-1.46) | 0.64(0.29-1.42) |
|  | | Middle | 1.32(0.85-2.03) | 0.76(0.48-1.19) | 0.61(0.28-1.36) |
|  | | Richer | 1.08(0.74-1.57) | 0.95(0.71-1.27) | 0.93(0.65-1.33) |
|  | | Richest | 1.00 (referent) | 1.00 (referent) | 1.00 (referent) |
| Pregnant/breast-feeding | | | 0.96(0.70-1.30) | 0.95(0.71-1.27) | 1.31(0.92-1.86) |
| No iron in pregnancy | | | 1.16(0.80-1.68) | 1.25(0.92-1.72) | 1.15(0.90-1.48) |
| ***Location*** | | |  |  |  |
|  | Rural | | 1.71(1.17-2.49)** | 1.21(0.75-1.96) | 0.61(0.32-1.16) |
| ***Province*** | | |  |  |  |
|  | Manicaland | | 1.00 (referent) | 1.00 (referent) | 1.00 (referent) |
|  | Mashonaland Central | | 1.32(0.76-2.30) | 0.70(0.45-1.08) | 1.40(0.94-2.08) |
|  | Mashonaland East | | 0.76(0.66-0.87)*** | 0.89(0.56-1.43) | 1.12(0.71-1.75) |
|  | Mashonaland West | | 1.36(1.17-1.58)*** | 0.71(0.42-1.21) | 1.60(1.01-2.53)* |
|  | Mat. North | | 1.11(0.83-1.49) | 0.64(0.29-1.38) | 1.52(1.00-2.30) |
|  | Matebeleland South | | 1.53(1.35-1.74)*** | 1.77(1.05-2.99)*** | 2.75(1.89-4.01)*** |
|  | Midlands | | 1.39(1.11-1.74)* | 1.37(088-2.11)*** | 1.63(1.06-2.51)* |
|  | Masvingo | | 1.51(1.27-1.80)*** | 0.71(0.41-1.26) | 1.10(0.70-1.73) |
|  | Harare | | 0.81(0.60-1.08) | 0.84(0.50-1.40) | 1.94(1.08-3.48)* |
|  | Bulawayo | | 1.64(0.48-0.86)*** | 0.83(0.46-1.49) | 1.70(0.99-2.92) |

*p<0.05, **p<0.01, ***p<0.001
